# Current Understanding of Leukocyte Phenotypic and Functional Modulation During Extracorporeal Membrane Oxygenation: A Narrative Review

**DOI:** 10.3389/fimmu.2020.600684

**Published:** 2021-01-08

**Authors:** Katrina K. Ki, Jonathan E. Millar, Daman Langguth, Margaret R. Passmore, Charles I. McDonald, Kiran Shekar, Manu Shankar-Hari, Hwa Jin Cho, Jacky Y. Suen, John F. Fraser

**Affiliations:** ^1^ Critical Care Research Group, The Prince Charles Hospital, Brisbane, QLD, Australia; ^2^ Faculty of Medicine, University of Queensland, Brisbane, QLD, Australia; ^3^ Roslin Institute, University of Edinburgh, Edinburgh, United Kingdom; ^4^ Clinical Immunology and Allergy, and Sullivan Nicolaides Pathology, Wesley Hospital, Brisbane, QLD, Australia; ^5^ Department of Anaesthesia and Perfusion, The Prince Charles Hospital, Brisbane, QLD, Australia; ^6^ Adult Intensive Care Service, The Prince Charles Hospital, Brisbane, QLD, Australia; ^7^ Department of Intensive Care Unit, Guy’s and St Thomas’ Hospital NHS Foundation Trust, London, United Kingdom; ^8^ School of Immunology & Microbial Sciences, King’s College London, London, United Kingdom; ^9^ Department of Paediatrics, Chonnam National University Children’s Hospital and Medical School, Gwangju, South Korea

**Keywords:** leukocyte modulation, innate and adaptive immunity, extracorporeal membrane oxygenation technology, critically ill patients, extracorporeal membrane oxygenation

## Abstract

A plethora of leukocyte modulations have been reported in critically ill patients. Critical illnesses such as acute respiratory distress syndrome and cardiogenic shock, which potentially require extracorporeal membrane oxygenation (ECMO) support, are associated with changes in leukocyte numbers, phenotype, and functions. The changes observed in these illnesses could be compounded by exposure of blood to the non-endothelialized surfaces and non-physiological conditions of ECMO. This can result in further leukocyte activation, increased platelet-leukocyte interplay, pro-inflammatory and pro-coagulant state, alongside features of immunosuppression. However, the effects of ECMO on leukocytes, in particular their phenotypic and functional signatures, remain largely overlooked, including whether these changes have attributable mortality and morbidity. The aim of our narrative review is to highlight the importance of studying leukocyte signatures to better understand the development of complications associated with ECMO. Increased knowledge and appreciation of their probable role in ECMO-related adverse events may assist in guiding the design and establishment of targeted preventative actions.

## Introduction

Extracorporeal membrane oxygenation (ECMO) is a potentially lifesaving modality that supports critically ill patients with refractory cardiac and/or respiratory failure (1–5). Over the last decade, the use and number of ECMO centers have dramatically increased (6). Beyond providing support to the heart and lungs, ECMO is used as a cardiopulmonary resuscitation tool and a bridge to transplant. The modern era of ECMO, post 2007, introduced significant advances in circuit design and improved expertise in clinical management (7). Despite these improvements, adverse events remain common. ECMO is associated with an increased risk of complications such as exacerbated systemic inflammatory response syndrome (SIRS), infection, new organ dysfunction, and thrombosis (6, 8–10).

The main cause for these complications may be alteration of blood components by their contact with the foreign and non-endothelialized surfaces of the ECMO circuit (comprises of a pump and membrane oxygenator connected by circuit tubing). However, delineation of ECMO-related changes can be challenging due to the underpinning pathophysiology of the critically unwell patients. It is further complicated by variances in technology, exertion of non-physiological conditions, and intensive use of modulatory clinical resources during ECMO. Activation of peripheral pro-inflammatory responses and the hemostatic system (platelet and coagulation) are widely recognized in the setting of ECMO thus far (11, 12). Likewise, differences in the closely regulated and inter-dependent blood leukocyte subset (namely monocytes, neutrophils, B and T lymphocytes) profiles have also been evaluated.

**Figure 1:**
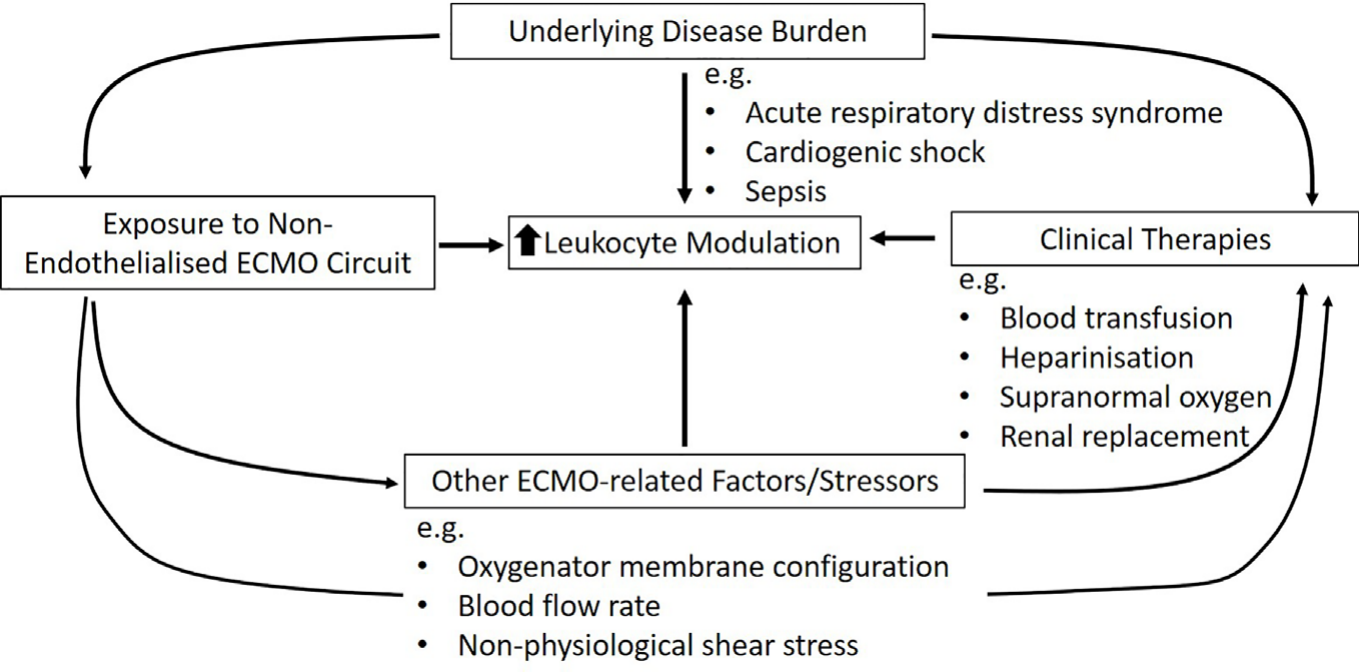
Summary of potential contributors to leukocyte modulation in critically ill ECMO patients.

Existing research reported alteration of leukocyte numbers, phenotype, and functions with non-specific activation when ECMO is used (**Tables 1** and **2**). A similar response has been observed in other extracorporeal circulatory modalities preceding ECMO, such as cardiopulmonary bypass (CPB), renal replacement therapy, and ventricular assist devices (VAD) (40–49). The sequelae linked to inappropriate leukocyte modulation can be detrimental. This is because leukocytes are multifaceted and play a critical role in the intricate communication between innate and adaptive immunity, regulation, and stimulation of inflammatory responses and self-tolerance (50), and interaction with platelets and endothelial cells that contribute to coagulation (51–55). These changes are also common in refractory cardiac and respiratory failure patients, triggered by the underlying disease that necessitated ECMO (56–59). Therefore, it is possible that the changes observed in these illnesses could be compounded by ECMO and associated factors/stressors (**Figure 1**).

**Table 1:** Clinical studies investigating changes in leukocyte numbers, phenotype, and functions in the context of ECMO.

Study (year, type) Study population (total n)	Reported indications	Pump system	ECMO membrane	Circuit tubing	ECMO config.	Summary of leukocyte-related outcomes	Ref
**Hocker et al.** **(1990, prospective obs.)** *Neonates (6)*	MAS, PPHN, RDS, AFA	Unknown	Silicone	Unknown	VA	Neutrophil and monocyte numbers reduced significantly during the initial 2 h of ECMO, and remained low until decannulation. Compared to monocytes, neutrophil recovery was slow. Lymphocyte numbers were unchanged. Neutrophil CD11b/CD18 expression (activation) increased during the initial 2 h of ECMO, and returned to pre-ECMO level after 6–24 h. No ECMO-related changes were observed in neutrophil phagocytic function, or monocyte HLA-DR expression and LPS response when compared to pre-ECMO.	(13)
**Zach et al.** **(1990, prospective obs.)** *Neonates (20)*	Unknown	Vortex	Silicone	Unknown	VA	Neutrophil and lymphocyte numbers reduced substantially after ECMO cannulation. Lymphocyte numbers began to recover on day 5, while neutrophil numbers remained low throughout the assessed 7 ECMO days.	(14)
**DePalma et al.** **(1991, prospective obs.)** *Neonates (25)*	PFC, CDH, RDS, PH	Roller occlusion	Silicone	Unknown	VA	Significant drop in overall lymphocyte counts after ECMO cannulation compared to pre-ECMO, and recovered by day of decannulation. No changes were observed in B lymphocyte numbers, and T lymphocyte subset numbers. Expression of activation CD25 and HLA-DR surface marker also unchanged.	(15)
**Plotz et al.** **(1993, prospective obs.)** *Neonates (10)*	MAS, CDH, RDS, PH, GBSP sepsis	Roller	Silicone	PVC	VA, VV	Leukocyte numbers dropped on the day of ECMO cannulation compared to pre-ECMO. Slow recovery was observed after 48 h, and returned to pre-ECMO levels post-cannulation.	(16)
**DePuydt et al.** **(1993, prospective obs.)** *Neonates (66)*	Unknown	Unknown	Unknown	Unknown	Unknown	Neutrophil phagocytosis and intracellular killing indices of ECMO neonates were significantly higher against *in vitro* candida stimulation, compared to healthy neonates. The effects were independent of ECMO duration.	(17)
**Fortenberry et al.** **(1996, prospective obs.)** *Neonates (15)*	PPHN, MAS, GSBP sepsis, CDH and more	Roller	Unknown	Unknown	VA, VV	CD11b expression on neutrophils peaked at 15 min of ECMO. Began to reduce after 30 min and dropped to below the pre-ECMO expression after 24 h. A similar trend was also observed for NE and IL-8 plasma levels. Authors correlated neutrophil activation with early pulmonary deterioration. However, no reported changes in CD62L expression, IL-6 and TNF-α levels.	(18)
**Kawahito et al.** **(1998, retrospective)** *Adults (16)*	Cardiogenic shock	Unknown	Unknown	Unknown	VA	Lymphocytopenia occurred in both survivors and non-survivors after ECMO. While lymphocyte numbers recovered after weaning in survivors, they remained low in non-survivors. This was correlated with infection-mediated mortality after weaning of ECMO reported in 90% of non-survivors.	(19)
**Hong et al.** **(2015, prospective obs.)** *Adults (23)*	Cardiogenic shock	Centrifugal	Hollow-fiber	Unknown	VA	Sharp reduction in overall lymphocytes and CD4^+^ T (helper) lymphocytes after 2 h of ECMO compared to when ECMO was being cannulated. Authors associated overall lymphocytes at 2 h, T helper lymphocytes at 6H, and overall T lymphocytes at 24 h with increased mortality, alongside a number of other markers.	(20)
**Ziemba et al.** **(2016, prospective obs.)** *Neonates, pediatrics (13)*	Unknown	Unknown	Unknown	Unknown	VA, VV	LPS-induced HLA-DR expression on monocytes decreased at initiation of ECMO compared to healthy controls. Paralleled by low LPS-induced TNF-α and PHA-induced IFN-γ and IL-10 release. Low HLA-DR expression persisted over the entire duration of ECMO, and proposed to increase risk for secondary infection while on ECMO.	(21)
**Liu et al.** **(2017, prospective obs.)** *Adults (51)*	ARDS ± pulmonary hypertension, arrhythmia, and more	Unknown	Unknown	Unknown	VA, VV	High IL-10 levels pre- and 24 h after initiation of ECMO were linked to a poorer prognosis, which was associated with slow or delayed recovery of CD14^+^CD16^+^ and CD14^+^TLR4^+^ monocytes and regulatory T lymphocyte numbers.	(22)
**Wilm et al.** **(2017, prospective obs.)** *Adults (41)*	Cardiac and respiratory failure	Unknown	PMP hollow-fiber	Unknown	VA, VV	Leukocyte adhesion onto oxygenators increased in younger (30–47 years old) patients with higher leukocyte counts and was reduced in older patients (61–71 years old). No negative effects were reported for either population.	(23)
**Bredthauer et al.** **(2017, prospective obs.)** *Adults (38)*	Cardiac and respiratory failure	Unknown	PMP hollow-fiber	Unknown	VA, VV	Leukocyte adhesion differs between two different ECMO systems. A greater coverage (88%) of small single-cell leukocytes was reported for Xenios AG Hilite7000LT membrane oxygenators compared to Maquet (-Getinge) PLS membrane oxygenators (33%). No significant difference in coagulation disorder was reported between systems.	(24)
**Francischetti et al.** **(2017, retrospective)** *Adults, pediatrics (166)*	Sepsis, ARDS, MAS, CHF and more	Unknown	Unknown	Unknown	VA, VV	Significant reduction in overall lymphocyte numbers after ECMO initiation was linked to mortality, alongside a number of laboratory values.	(25)
**Santiago-Lozano et al. (2018, Retrospective)** *Pediatrics (100)*	Sepsis, cardiac disease, respiratory failure	Unknown	Unknown	Unknown	VA	No changes in overall leukocyte numbers between ECMO patients with or without subsequent infection were observed. Paralleled by lack of changes in C-reactive protein. ECMO-related leukocyte reduction was observed, but not discussed within the publication.	(26)
**Ortega et al.** **(2019, prospective obs.)** *Neonates, pediatrics (25)*	Septic shock, cardiogenic shock, PPHN, CDH, MAS, cardiac arrest, respiratory failure and more	Centrifugal	Unknown	Unknown	VA, VV	Activated CD4^+^ T (helper) lymphocytes expressing CD161 reduced, and T-cell autoreactivity to CNS antigens increased with ECMO support compared to sick controls. Further, ECMO patients presented with acquired brain injury and cerebral autoregulation impairment were reported with increased CD8^+^ (cytotoxic) T lymphocyte and B lymphocyte selective autoreactivity to CNS antigens. Increased CD161^+^ cytotoxic T, B, and NKT lymphocytes in these patients were also observed, paralleled by significant IL-6 and IL-8 plasma levels. No significant changes were observed in other studied leukocyte populations between the patient groups.	(27)
**Sargin et al.** **(2019, retrospective)** *Adults (119)*	Post-cardiotomic shock	Unknown	Unknown	Unknown	VA	A significant NLR increase in the first 3 days of ECMO was observed in patients who developed renal failure. These ECMO patients had a comparatively higher NLR to those who did not.	(28)
**Han et al.** **(2020, prospective obs.)** *Unknown (57)*	Unknown	Unknown	Unknown	Unknown	Unknown	ECMO reduced monocyte capacity to secrete IL1β, IL-6, and TNF-α compared to healthy controls.	(29)

A structured search of EMBASE and PubMed/MEDLINE for all relevant publications was conducted. Search terms were “ECMO” OR “extracorporeal membrane oxygenation” OR “ECLS” OR “extracorporeal life support” OR “artificial heart/lung” AND “leukocytes” OR “white blood cells” OR “immune response.” Studies published specifically to report on leukocyte modulation during ECMO were included (see **Supplementary Material** for detailed literature search strategy).

AFA, amniotic fluid aspiration; ARDS, acute respiratory distress syndrome; CD, cluster differentiation; CDH, congenital diaphragmatic hernia; CHF, chronic heart failure; CNS, central nervous system; GBSP, Group B Streptococcus;, H, hours; HLA-DR, human leukocyte antigen-DR isotype; IL, interleukin; IFN, interferon; LPS, lipopolysaccharide; MAS, meconium aspiration syndrome; Min, minutes; NKT, natural killer T lymphocyte; NLR, neutrophil-to-lymphocyte ratio; Obs., observational; PHA, phytohemagglutinin; PLS, permanent life support; PFC, persistent fetal circulation; PH, pulmonary hypoplasia; PMP, polymethylpentene; PPHN, persistent pulmonary hypertension of the newborns; PVC, polyvinylchloride; RDS, respiratory disease syndrome; VA, veno-arterial; VV, veno-venous.

**Table 2:** Experimental studies investigating changes in leukocyte numbers, phenotype, and function in the context of ECMO.

Study (year)	Model (species),System rep.(total n)	Pump system	ECMO membrane	Circuit tubing	Summary of leukocyte-related outcomes	Ref
**Bergman et al. (1994)**	*Ex vivo* (human), Neonatal (16)	Centrifugal pump	Silicone	PVC	Overall leukocyte number and deformability reduced significantly during the 72 h of ECMO compared to pre-ECMO levels. Leukocyte adhesion and clogging rate to the oxygenator membrane also increased.	(30)
**Skogby et al.** **(1998)**	*Ex vivo* (human), Neonatal (16)	AREC *vs.* centrifugal pump	Hollow-fiber (type unknown) *vs.* Silicone	Silicone *vs.* PVC	Two perfusion systems were compared: AREC perfusion system had lower neutrophil loss and CD11b expression during the 24 h of ECMO compared to centrifugal system. No differences were observed for expression of CD11a and ICAM-1, or plasma levels of cytokines and chemokines (IL-1β, IL-6, IL-8) between systems.	(31)
**Graulich et al. (2000)**	*Ex vivo* (human), Neonatal (6)	Nonocclusive roller pump	Silicone	PVC	Early activation of both neutrophils and monocytes was characterized by increased CD18 expression and CD62L shedding after 2–4 h of ECMO compared to baseline. After 8 h, expression of CD18 returned to baseline level. While the reduced CD62L expression, and increased plasma NE level persisted. No leukocyte loss, or lymphocyte phenotypic changes observed.	(32)
**Adrian et al.** **(2003)**	*Ex vivo* (human), Neonatal (8)	Roller pump	Silicone	Unknown	Three groups were compared: No S-nitrosglutenione (GNSO), low and high GNSO doses (no differences found). Regardless of GNSO, total leukocyte and neutrophil numbers reduced during the 24 h of ECMO. Increased CD18 and CD11b expression was observed at initiation of ECMO and reduced after 3 h in all groups. GSNO did not dampen ECMO-mediated inflammatory responses.	(33)
**McILwain et al. (2010)**	Animal (porcine), Neonatal (16)	Centrifugal pump	Microporous PP Hollow-fiber	Unknown	Increased CD18, CD35, CD62L, and CD11b expression on neutrophils following 2 h of VA-ECMO in comparison to sham controls. Leukocyte infiltration also elevated resulting in damage in both lungs and intestine after 8 h of ECMO. Paralleled by increase in plasma IL-1β, IL-6, IL-8, and TNF-α levels, and tryptase activity (associated with degranulation of mast cells).	(34)
**Rungatscher et al. (2015)**	Animal (rat), Unknown (20)	Roller pump	Hollow-fibee (type unknown) *vs.* No oxygenator	Unknown	The effect of extracorporeal circuit with and without oxygenator was compared. p38 MAPK and NF-κB phosphorylation (inflammatory pathways) significantly upregulated in mononuclear leukocytes and granulocytes after 1H of extracorporeal circulation in the presence of an oxygenator (“ECMO”). Paralleled by the rise in IL-6, TNF-α, and NE plasma levels. An increased neutrophil infiltration and severe lung injury scores was also reported.	(35)
**Passmore et al. (2016)**	Animal (ovine), Adult (43)	Centrifugal pump	PMP hollow-fiber	PVC	ECMO significantly reduced leukocyte numbers in circulation after 24 h in an ovine model with pre-existing lung injury compared to those with injury alone. The combination also promoted monocyte/macrophage and neutrophil MMP2 and MMP9 expression in lung tissues, and increased lung infiltration. All aligns with the increased lung oedema and inflammatory response reported.	(36)
**Ki et al.** **(2019)**	*Ex vivo* (human), Adult (10)	Centrifugal pump	PMP hollow-fiber	PVC	Two ECMO flow rates were compared: high (4 LPM) and low 1.5 (LPM). Both models showed ~50% reduction in monocyte numbers over 6 h of circulation, but not others. A rapid release of NE and MPO by activated neutrophils over time during high flow compared to low flow. Paralleled by elevated plasma levels of IL-1β, IL-6, and TNF-α. However, no differences in leukocyte subset activation (CD18, CD11b, CD66b, HLA-DR, CD25, and CD80 expression) were observed between groups.	(37)
**Zhang et al. (2019)**	Animal (porcine), Unknown (24)	Centrifugal pump	PMP hollow-fiber	PVC	Compared to conventional cardiac arrest post-resuscitation method, ECPR (VA-ECMO) increased spleen CD4^+^ T (helper) lymphocytes and CD4^+^/CD8^+^ T lymphocyte ratio, and enhanced T lymphocyte proliferation and low apoptotic rate. Paralleled by increased IL-2, IL-4 and IFN-γ levels and reduced ROS production in the spleen. All associated with increased survival.	(38)
**Meyer et al.** **(2020)**	*Ex vivo* (human), Neonatal (15)	Roller pump	Microporous PC hollow-fiber	Unknown	Three ECMO flow rates were compared: low (0.3 LPM), nominal (0.5 LPM), and high (0.7 LPM). Greatest leukocyte-derived EV released at high flow followed by low flow, peaked at 2–4 h of ECMO. Expression of tissue factor on leukocytes and their EV exhibited a similar trend, may be contributing to occlusive thrombosis. Akin to static control, nominal flow remained unchanged.	(39)

A structured search of EMBASE and PubMed/MEDLINE for all relevant publications was conducted. Search terms were “ECMO” OR “extracorporeal membrane oxygenation” OR “ECLS” OR “extracorporeal life support” OR “artificial heart/lung” AND “leukocytes” OR “white blood cells” OR “immune response.” Studies published specifically to report on leukocyte modulation during ECMO were included (see **Supplementary Material** for detailed literature search strategy).

AREC, Assistence respiratoire extracorporelle; CD, cluster of differentiation; CD62L, L-selectin; ECPR, extracorporeal cardiopulmonary resuscitation; EV, extracellular vesicles; GNSO, S-nitrosglutenione; IFN, interferon; IL, interleukin; LPM, liters per minute; MMP, matrix metalloproteinase; MPO, myeloperoxidase; NE, neutrophil elastase; NF-κB, nuclear factor kappa-light-chain-enhancer; p38 MAPK, p38 mitogen-activated protein kinase; PC, phosphorylcholine; PVC, polyvinylchloride; PMP, polymethylpentene; PP, polypropylene; ROS, reactive oxygen species; System rep, system represented; TNF, Tumor necrosis factor; VA, Veno-arterial; VS., Versus.

Currently, there is limited knowledge regarding leukocyte phenotype and functions during ECMO, including whether these changes have attributable mortality and morbidity. Much of what we know about the effects of ECMO on leukocytes and the immune system is based on changes in the inflammatory response (11), as well as prior knowledge from CPB (41–43, 60, 61). Given the pivotal contribution leukocytes can have in both immune and hemostatic complications, better characterization of their ECMO-related phenotypic and functional signatures could be beneficial. In particular, the capacity to separate the immunologic signatures between the patients’ leukocyte-modulating primary disease burden and ECMO, could facilitate patient risk stratification and prognosis. Additionally, by optimizing ECMO circuit design we may lessen its effects on leukocytes to help reduce poor outcomes.

This review aims to provide a summary of both published clinical and experimental evidence on the effects of ECMO on the innate and adaptive leukocyte numbers, phenotype, and functions, and to highlight their potential role in ECMO outcomes. In addition, the influences of different ECMO circuit designs and factors (clinical therapies and ECMO-related stressors) that may further contribute to the occurrence of leukocyte modulation will be discussed. Finally, we address the limitations and future directions on this Research Topic.

## ECMO as a Second “Hit” to Leukocyte Pathophysiology in Critically Ill Patients

**Figure 2:**
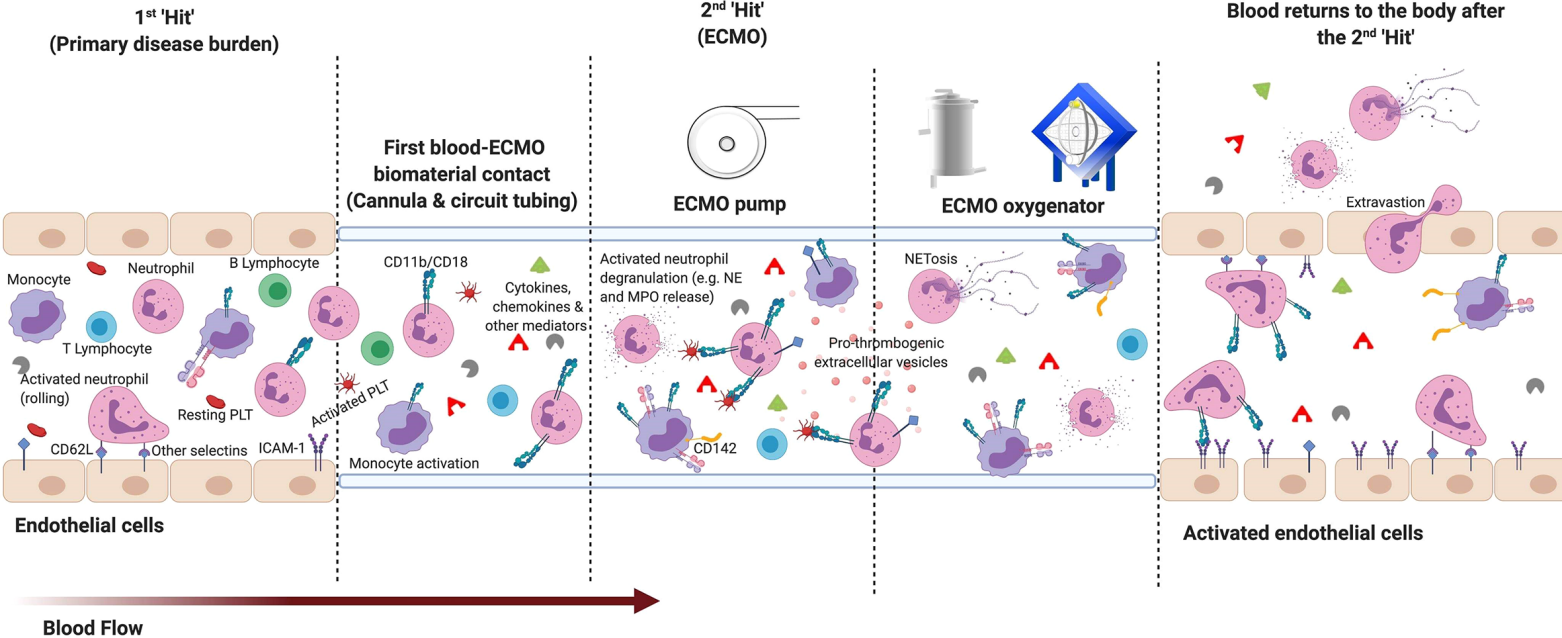
The two “hit” ECMO model: Basic overview of early leukocyte modulation during extracorporeal membrane oxygenation (ECMO). *1^st^ hit:* Immune modulation from the patient’s primary disease burden. *2^nd^ hit:* The inflammatory response to ECMO is proposed to activate upon contact of blood with the non-endothelialized surface of the ECMO circuit and the non-physiological conditions generated. ECMO-mediated promotion of pro-inflammatory cytokines and chemokines, and other mediators (e.g. complement products) from non-physiological exposure, contact and cellular activation can lead to further activation of leukocyte subpopulations. During early ECMO, innate immune neutrophils and monocytes become the first to activate—consistently reported with an increased expression of surface integrins cluster differentiation (CD)11b and CD18. Alongside L-selectin (CD62L), these modulated integrin expressions potentiate an increase in leukocyte adhesion to the membrane oxygenator, as well as activated endothelium (e.g. increased Intercellular Adhesion Molecule (ICAM)-1) expression) and platelets [PLT; e.g. increased P-selectin glycoprotein ligand-1 (PSGL) and glycoprotein Ib alpha polypeptide (GIbα) expression]. As blood continues to circulate through the ECMO circuit: monocyte expression of tissue factor (CD142), neutrophil degranulation, neutrophil extracellular trap (NET)osis, pro-thrombogenic extracellular vesicle release and activation of downstream lymphocytes are also upregulated. All of which can contribute to the heightened systemic inflammatory response syndrome (SIRS), leukocytopenia, infection, end-organ damage, and thrombosis frequently reported in patients. (Figure created with BioRender.com).

Leukocyte dysfunction is common in critical illnesses such as acute respiratory distress syndrome (ARDS), cardiogenic shock, and sepsis (56–58, 62), which are also frequent indications for ECMO support. As changes in the functional immune response cannot be sufficiently characterized by the widely researched inflammatory mediators alone, due to their short half-life and exclusive origin, cellular profiling may be beneficial. Leukocyte numeric, phenotypic and functional alterations have long been recognized as important indicators of inflammation and predictors of poor outcomes. These changes are associated with increased risk of secondary infection, end organ injuries, mortality, and a prolonged length of hospital stay in critically ill patients (56–59, 63, 64). Studies have suggested that veno-arterial (VA; heart and/or lung support) and veno-venous (VV; lung support) ECMO further dysregulates the leukocyte pathophysiology in refractory neonates, pediatrics, and adults. These applications act as a second “hit” to the immune system (**Figure 2**).

In this section, we provide an overview of current studies (**Tables 1** and **2**) published specifically to report on the effect of ECMO on leukocytes (see **Supplementary Material** for detailed literature search strategy). It will focus on the four major peripheral leukocyte subsets of the innate and adaptive immunity: neutrophils, monocytes, T and B lymphocytes.

### Impact on the Innate Immune Leukocytes

Neutrophil and monocyte profiles are the most studied peripheral leukocytes in ECMO. It is likely due to their abundant expression, and critical role in the initiation of rapid immune response and direction of downstream adaptive immunity. During neonatal and adult ECMO, a dramatic fall of neutrophils and monocytes is consistently described. It can occur within 2 h of ECMO cannulation (13, 14, 22). In contrast to CPB where the number of leukocytes increased during open heart surgeries (40, 43), leukocytopenia has long been reported in ECMO (16, 25). Differences could be associated with the pathophysiology between ECMO and CPB patients, and the involvement of other immune triggers (e.g. induction of surgical trauma and ischemic insults in cardiac surgery patients). Studies also correlated slow or delayed recovery of monocytes with worsen prognosis and mortality in both VA and VV ECMO patients (22). Specifically, low number of cluster differentiation (CD)14^+^CD16^+^ and CD14^+^ toll-like receptor (TLR)4^+^ monocyte subsets have been linked to non-survivors (22).

In comparison, the changes in neutrophil and monocyte numbers were variable in *ex vivo*. While the clinical trend of reduction was reported by Adrian et al., this was not observed by Graulich et al. and partially observed by Ki et al. (32, 33, 37). The discrepancies between the outcomes could be rationalized by the utilization of different ECMO technology, model setup, and blood flow rates. In addition, the lack of endothelial lining in the current models may contribute to these differences between *ex vivo* studies, as well as *ex vivo* and clinical studies. In response to the continuous exposure to non-physiological conditions (e.g. flow rates and associated shear stress) and significant release of inflammatory mediators during ECMO (16, 18, 34, 35, 37, 39, 65–67), cells of the endothelial lining are likely to convert into an activated state (68, 69). This can subsequently elevate leukocyte adhesion [from phenotypic modulation (13, 17, 18, 34–36)] onto either the artificial surface of the ECMO circuit (23, 24, 70) or the endothelium itself (68, 69), possibly acting as a consistent regulator of cell attachment *in vivo*.

Furthermore, current animal and human research have characterized a number of leukocyte phenotypic profiles associated with ECMO (see **Supplemental Table 1** on discussed leukocyte markers). Activation of neutrophils during ECMO is predominantly identified by the increased expression of CD11b and CD18 in both clinical and experimental ECMO settings (13, 17, 18, 31–36). Similar phenotypic modulation of neutrophils is reported in CPB patients post-operation (43, 71), but their expression begins to downregulate after 3–4 h of ECMO and can persist for a prolonged duration (18, 32, 34). This is also observed in other innate immune cells (32), which could be associated with an increased adhesion or binding with ligands as part of the activation process during ECMO. CD11b and CD18 are integrins essential to regulating adhesion and migration of leukocytes to mediate the inflammatory response. Modulation of these molecules expressed on the surface of leukocytes, including neutrophils, can have an impact on their crucial role in trans-endothelial and trans-epithelial migration to areas of inflammation as part of host immune defense (36, 72). However, the effect of ECMO on L-selectin (CD62L), another key adhesion molecule, remains debatable. Three studies independently reported either a reduced, augmented, or no change in expression of L-selectin at the initial hours of ECMO (18, 32, 33). Observed discrepancies may be associated with the differences between human, porcine and *ex vivo* ECMO. Activation of neutrophils is also characterized by degranulation. During ECMO, neutrophil elastase (NE) and myeloperoxidase (MPO) levels rise significantly within 2–4 h alongside other inflammatory mediators (31, 32, 34, 37). NE and MPO are pro-inflammatory granules released into the extracellular space by activated neutrophils for clearance of Gram-negative bacteria and mediate anti-microbial activities (73). It is part of an emerging concept for neutrophil function, the formation of neutrophil extracellular traps (NETs).

Alongside neutrophils, monocyte phenotype that tightly governs their functions is also altered as seen in valve replacement patients exposed to CPB. Monocytes become activated with increased CD11b, but the surface expression of human leukocyte antigen (HLA)-DR was significantly lower compared to the preoperative state (43). Indeed, such modulation is witnessed in experimental and clinical ECMO (21, 32). Monocytes expressing TLR4, an essential receptor in the recognition of bacterial pathogens, were also low during VV-ECMO. Authors speculated that it is caused by intensive endocytosis of CD14/TLR4 due to rampant damage-associated molecular pattern molecules from the pre-existing ARDS and further exacerbating by the immunological imbalance mediated by ECMO (22). These profiles are also observed in infected and septic patients (58, 63), and some are linked to mortality (22, 74).

## Impact on the Adaptive Immune Leukocytes

T and B lymphocytes are essential to the adaptive immune system: if they become dysregulated, the capacity of the immune system to distinguish self from foreign and provide specialized immune defense can be hindered. Reduction in the overall lymphocyte numbers during ECMO, and the impediment of their recovery post-weaning is associated with mortality in patients (14, 15, 19, 20). A significantly lower T regulatory lymphocyte number (a subset of T lymphocytes) has been further identified in deceased patients on day 3 of ECMO, coupled with high anti-inflammatory interleukin (IL)-10 (22). A similar reduction was evident in valve replacement surgery patients using CPB (43). More recent publications have examined the potential of neutrophil-to-lymphocyte ratio (NLR) as a predictor of poor prognosis and survival in post-cardiotomic shock patients supported with ECMO. NLR is a ratio determined by dividing neutrophil absolute counts by lymphocyte absolute counts and is an indicator of perturbed leukocyte homeostasis. ECMO support is reported to increase the NLR values, and patients with high NLR have an increased risk for renal complications and mortality compared to those with a lower ratio (28). In CPB, higher NLR is associated with longer extubation time and a longer duration of ICU stay, consistent with ECMO (75). The prognostic capacity of NLR is further demonstrated across various critical illnesses (56, 76, 77).

Unlike the innate immune cells, phenotypic profiling of T and B lymphocyte modulation during ECMO is infrequent. An *ex vivo* ECMO study reported no changes in CD18 and CD62L expression in overall lymphocyte population (32). While CD161 expressing T helper lymphocytes were downregulated in both VA- and VV-ECMO neonates and pediatrics, changes were not observed for cytotoxic T or B lymphocytes. There remains a need for more studies to provide further indications of the role ECMO may have on the adaptive immune response. Future phenotypic research may also benefit from investigating other key subsets such as T helper 1, 2, and 17 lymphocytes over the overall lymphocyte or T helper population. Each T helper lymphocyte subset have specialized function and response required for differential adaptive immune response, thus, changes of the subsets could be overlooked when assessing the wider population.

Taken together, innate and adaptive leukocyte numbers can be a powerful prognostic tool, and development of a more comprehensive database including a larger heterogenous ECMO population could prove advantageous. However, the data generated cannot inform delineation of changes mediated by pre-existing disease and ECMO, or targeted future therapies. Based on the indicated profile of these leukocytes, increased surface molecules for adhesion and activation are associated with leukocytopenia. Therefore, characterization of leukocyte phenotype during ECMO would be complementary and important.

## What Are the Causes of ECMO-Related Leukocyte Modulation?

### ECMO Technology (Circuit Designs)

Direct contact of blood with the foreign surface of the ECMO circuit is widely accepted as a consequence of modulated blood physiology. While advances in ECMO technology have improved circuit biocompatibility and reduced activation of the coagulation system, significant immune changes persist, potentially contributing to SIRS, infection, new organ dysfunction, and thrombosis.

#### Blood Pumps

Blood pumps are a crucial component for extracorporeal blood circulation within the ECMO module. CPB and ECMO studies established the benefits of newer modified centrifugal pumps over roller pumps on blood damage reduction (78–80). However, no direct comparative investigation has been performed on ECMO specific to leukocyte modulation to accurately discern the differences. Previous CPB studies have demonstrated lower leukocyte loss, alongside reduced hemolysis, inflammatory response, and platelet activation when using centrifugal pumps (79). ECMO is assumed to follow a similar trend, and changes may lie largely with the oxygenator.

#### Oxygenators

Another major technological evolution of ECMO after 2007, was the implementation of polymethylpentene (PMP) hollow-fiber oxygenator membranes required for oxygenation. Early generations used bubble and silicone-based spiral coil membrane oxygenators that resulted in a significant drop in overall circulating leukocyte numbers in comparison to hollow-fiber membrane oxygenators. ECMO using The Kolobow silicone oxygenator membrane also reported increased leukocyte loss, deformability, and clogging rate to the oxygenator *ex vivo*, compared to hollow-fiber (30, 31). Observed enhancement for hollow-fiber membranes are associated with dampened activation of neutrophils characterized by the lower expression of CD11b adhesion molecule, and NE and superoxide anion release (30, 81).

Hollow-fiber membrane oxygenators are designed for a longer use duration (days to weeks) and have enhanced biocompatibility, however, modulatory effects are still reported (**Tables 1** and **2**). High cellular and coagulation factor deposits on PMP membrane oxygenators are observed (23, 24, 70). The immune modulatory influence of extracorporeal circuit with and without an hollow fiber oxygenator was compared using a rat model (35). Two major inflammatory signaling pathways of leukocytes, p38 mitogen-activated protein kinase (MAPK) and Nuclear factor kappa B (NF-κB) phosphorylation, were significantly upregulated after 1 h of extracorporeal circulation. An increase in severe lung injury scores was also reported, while no significant leukocyte changes or organ injury were observed in the absence of an oxygenator. Guided by reduced leukocyte and platelet adhesion profile, Obstals et al. proposed that optimization of the membrane surface using other non-thrombogenic polymer brushes could be beneficial (82).

Although the PMP hollow-fiber membrane oxygenators are now ubiquitous in the clinical setting, many different systems and manufactures are available. The degree of leukocyte and other cellular modulation mediated is likely to be different between centers and countries, in part, for this reason. As Bredthauer et al. have demonstrated, there are clear differences in leukocyte adhesion, as well as vWF deposition on PMP hollow-fiber oxygenator membranes between commercially available systems such as the Maquet(-Getinge) permanent life support (PLS) and Xenios AG Hilite LT (24). Variances in how a membrane is configured within the oxygenator can also influence blood flow rates and ultimately cellular and humoral responses. This is supported by the CPB literature, differential cellular deformability were observed when different oxygenators of the same microporous polypropylene hollow-fiber membrane were compared (83). However, careful consideration into the influences of other factors such as age, gender, and the patients’ underlying disease burden are essential for future studies comparing the potential impact of the different technologies (23, 24, 70).

### Other ECMO-Associated Factors

Beyond ECMO technology, there are other common factors associated with ECMO that are likely to contribute to the leukocyte and blood component profile reported in patients. These factors include ECMO flow rates and the associated non-physiological shear stress exerted during extracorporeal circulation. However, current knowledge in this research area is very limited.

Two recently published *ex vivo* studies reported ECMO flow rates and the mediated non-physiological shear stress as potential influencing factors (37, 39). During continuous ECMO blood flow, wall shear stress within the rigid ECMO circuit increases to non-physiological levels as blood flow elevates. As illuminated by CPB, VAD, and fluid dynamics studies, variation in these parameters can differentially influence leukocyte phenotype, function, migration, and deformability (47, 84–88). At high adult and neonatal ECMO flow, significant increases in neutrophil activation, characterized by a rise in plasma neutrophil granules (NE and MPO), and leukocyte-derived extracellular vesicles were observed during 6 h of ECMO, in comparison to low flow. An upregulated expression of tissue factor on these extracellular vesicles and leukocytes was also reported, which could contribute to ECMO-related inflammation and thrombosis. The findings were further supported by a rapid increase of pro-inflammatory cytokines and chemokines, resembling SIRS (37). However, the observed differences between high and low flow rates were not detected experimentally for leukocyte activation phenotype (37), and clinically for hemolysis (89). These may be attributed to the absence of an underlying primary disease burden and an endothelium, as well as the use of cannula and the timing of blood collection *ex vivo.* In addition, for each oxygenator design and size there is likely an optimal flow for different cellular components that differentially minimizes non-physiological shear stress and damage, as seen in Meyer et al. study (39). Further animal and clinical research is required to validate the *ex vivo* effect of ECMO flow rate specific to leukocyte modulation.

Of note, the ECMO patient population is extremely diverse. This makes it challenging to characterize the effect of ECMO-related leukocyte modulation and to discern targets for intervention. In addition to their differential ECMO support, medications, anti-coagulant strategies, and premorbid health, patients are also exposed to different stressors and therapies. Therefore, investigations into other frequently used therapies with known leukocyte modulatory effects are also warranted as they are resource intensive.

## What Are the Implications of Leukocyte Modulation in ECMO Patient Outcomes?

### Infection

**Figure 3:**
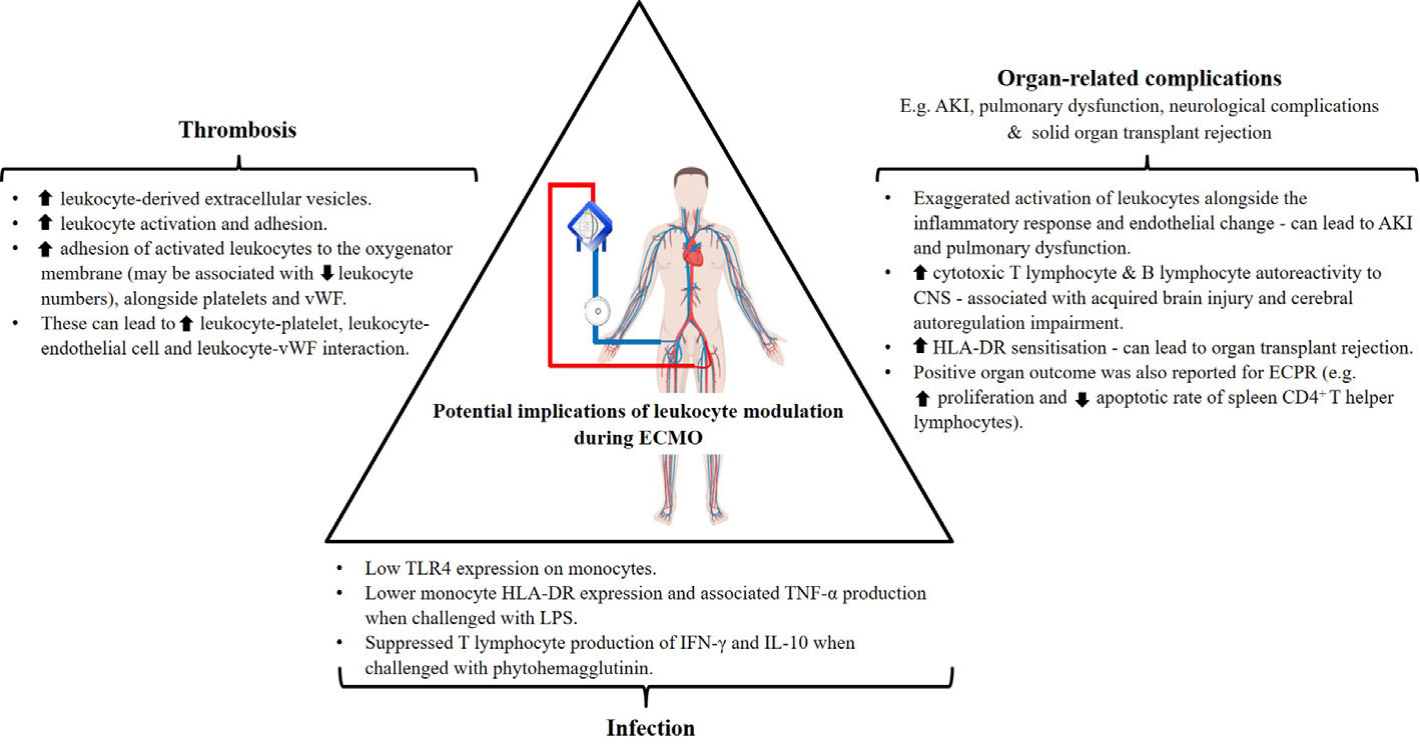
Potential implications of documented leukocyte modulation in ECMO patient outcomes. *human leukocyte antigen (HLA)-DR, tumor necrosis factor (TNF)-α, interferon (IFN)-γ, interleukin (IL)-10, acute kidney injury (AKI), toll-like receptor (TLR)-4, lipopolysaccharides (LPS), von Willebrand factor (vWF)* (Figure partially created with BioRender.com).

Development of infection is witnessed across various extracorporeal supports despite differences in the underlying primary disease burden in patients. Incidences are reported in cardiac patients subjected to open-heart cardiac surgery (90) and VAD implant (91), in addition to ECMO (with 3.5–64% infection rate) (8). These are often attributed to the exposure of wounds from surgeries, driveline insertion or cannulation. Santiago-Lozano et al. demonstrated that overall leukocyte numbers alone were unable to identify patients who will develop infection following VA-ECMO cannulation (26), and investigation into leukocyte subsets may provide additional information. Further phenotypic and functional immune studies revealed the potential underlying role of acquired immunoparalysis in the occurrence of infection in these patient cohorts (**Figure 3**).

Monocytes and other antigen presenting cells (e.g. dendritic cells) express an abundant level of HLA-DR, which is used as a means of assessing innate immune function, as well as monocyte activation. During an infective episode, upregulation of HLA-DR expression on monocytes is anticipated to present antigens to lymphocytes, eliciting an immune response. Ziemba et al. have shown that upon endotoxin stimulation, monocyte HLA-DR expression and associated tumor necrosis factor (TNF)-α production were lower following ECMO than healthy controls (21). Low TLR4 expression on monocytes observed in non-survivors during early ECMO support may play a role in its lack of response, as it is a key pattern recognition receptor for endotoxin (22). Recently, Han et al. also proposed that reduced monocyte function is associated with increased CD71^+^ (transferrin receptor) immature erythrocytes found during ECMO, mediating an inhibitory effect (29). Upon depletion of these immune modulatory erythrocytes, beyond their traditional role, monocyte function was retained. In addition, ECMO patients are reported with suppressed T lymphocyte production of interferon (IFN)-γ and IL-10 following *ex vivo* phytohemagglutinin stimulation (21). These are consistent with Beshish et al. definition of innate immunoparalysis mediated by ECMO (92). The observed changes are also in line with profiles associated with sepsis, and nosocomial infections in post-CPB cardiac surgery and VAD implanted patients (40, 41, 49).

However, it remains speculative whether the lack of response from leukocytes is a result of ECMO use or the disease burden itself without a comparison to pre-ECMO response or sick controls. ECMO can be used to support patients who may already be immunoparalyzed such as those with sepsis and unsuccessful weaning from CPB, as shown in an earlier neonatal study (13). On the contrary, DePuydt and colleagues also reported enhanced candidacidal activity (phagocytosis and intracellular killing) in neonates (17). Differences may be associated with the variances between ECMO technology, duration, indication as well as the differential pathways of activation by fungi *versus* bacteria. Additional studies are necessary to determine whether the increased risk of infection in some patients is associated with the inability of leukocyte subsets to respond appropriately when immune challenges arise.

### Organ-Related Complications

Acute kidney injury (AKI) affects up to 60% of ICU patients and is associated with mortality rates of between 15 and 60% (93). It is also the most common organ complication associated with ECMO, resulting in up to 30–45% of patients requiring renal replacement therapy while supported by this modality (6, 9). Despite extensive investigation in the setting of CPB, and improved ECMO circuit design to reduce hemolysis, AKI still occurs. The cause of AKI is complex, making it difficult to discern the relative contributions of the patients underlying pathology from ECMO itself. Currently there is no evidence to implicate ECMO as an independent risk factor for AKI. Two general phenomena have been proposed: ischemia- or inflammation-mediated insult (94). If ECMO support is inadequate for delivery of oxygen and to support metabolic demands, ischemic insults can occur, triggering a cascade of immunologic consequences and vasoconstriction. Alternatively, it can occur due to overwhelming activation of the systemic inflammatory response, leukocytes and endothelial cells, as seen in critically ill patients (56–59) and in those supported with ECMO (18, 32–34). The aberrant immunologic response can result in hyperdynamic states that also lead to AKI (94). Similar phenomena were also correlated with the induction of pulmonary dysfunction (34–36), seen in 1–8% of ECMO patients (6) (**Figure 3**).

In addition to AKI and pulmonary dysfunction, the influence of ECMO-related leukocyte modulation to potentiate other organ-related changes is proposed. Ortega and colleagues observed an increased T lymphocyte selective autoreactivity to the central nervous system components when ECMO support is used compared to sick controls (27). Further sub-analysis of the ECMO patient group revealed elevated cytotoxic T lymphocyte and B lymphocyte autoreactivity to central nervous system components in those with acquired brain injury and cerebral autoregulation impairment (27). These changes may have contributed to the subcategories of neurological complications reported by the current Extracorporeal Life Support registry (6). In contrast to other studies, Zhang et al. observed improved organ outcomes following VA-ECMO when used as extracorporeal cardiopulmonary resuscitation (ECPR) for post-resuscitation in a porcine model of cardiac arrest (38). VA-ECMO provided better stabilization of hemodynamics, tissue perfusion and oxygenation compared to the traditional approach. It was found to enhance the function of the spleen, unlike conventional management. The spleen is a T-lymphocyte-rich immune reservoir essential to immunological responses, and is a commonly affected organ following cardiac arrest and traditional post-resuscitation methods. Use of ECPR was able to reduce reactive oxygen species production and mitochondrial dysfunction, which contributed to enhanced proliferation and low apoptotic rate of spleen CD4^+^ T helper lymphocytes (38).

To date, there is insufficient mechanistic and clinical data to clearly delineate if the occurrence of organ dysfunction is ECMO-related or simply a secondary progression to the primary disease burden necessitating ECMO. Given the pivotal involvement of leukocytes at the initiation phase of organ injury, further dedicated mechanistic studies should be considered. This may inform a new capacity to delineate the two, and enable clinicians to predict, treat, and ameliorate the clinical sequelae. Furthermore, ECMO has been reported to increase HLA-DR sensitization in solid organ transplant patients (95, 96). As more ECMO is being used for transplantation, a better understanding of the role of ECMO-related leukocyte modulation in this setting is also necessary.

### Thrombosis

Thrombosis is another serious complication of ECMO with the highest incidence recorded in neonates affecting up to 37.5% of patients (6, 10). Despite better anti-coagulation strategies using unfractionated heparin and development of heparinized circuitry, thrombosis still occurs. While the role of activated leukocytes and their upregulated expression of adhesion molecules in coagulation is acknowledged (11) (**Figure 3**), there is very little existing research in the setting of ECMO in comparison to the classic pathways. One study demonstrated increased production of both leukocyte- and platelet-derived pro-thrombogenic factors during ECMO circulation (39), as seen in VAD patients (48). In particular, increased tissue factor expression was reported on activated leukocytes and leukocyte-derived extracellular vesicles, acting as the activator of the tissue factor pathway for coagulation. Adhesion of these activated leukocytes to the oxygenator membrane, alongside platelets, and procoagulant factors such as von Willebrand factor (vWF), has also been hypothesized to further stimulate clot formation. A recently published study associated high level accumulation of VWF and other cellular deposits on the membrane oxygenator with elevated SOFA scores, severe thrombocytopenia, and persistent liver dysfunction in ECMO patients (70). Exposure of blood to extracorporeal circuitry and non-physiological conditions (increased shear stress), as seen in VADs and ECMO (97, 98), can also lead to alterations in vWF conformation and loss of normal pro-coagulant properties resulting in the development of acquired von Willebrand syndrome (99). In the other studies, no differences in the hemostatic parameters and clinical outcomes were observed between patients with more or less cellular deposits (23, 24). Nonetheless, the authors correlated higher deposition with younger patients (35 *versus* 61 years old) who had greater pre-ECMO leukocyte counts (23), and the oxygenator manufacturer/design used (24). Additional investigation into neonates and pediatrics, an even younger and thrombosis-prone population, may provide different insights. Furthermore, Bredthauer and colleagues showed that the frequently required anticoagulants used for ECMO inhibits neutrophil migration and chemotactic migration efficiency (100). The inhibitory effect of heparin and argatroban have been proposed to not only modulate neutrophil function but may also increase adhesion onto the membrane oxygenator to prompt clot formation.

Thrombosis can also occur upon adhesion of activated leukocytes to platelets and endothelial cells, two of the major players of coagulation. Complexes of leukocyte integrin (CD11b/CD18) with platelet adhesion molecules (P-selectin glycoprotein ligand-1 and glycoprotein Ib alpha polypeptide) are known to regulate thrombosis (51). The interaction has been suggested to be a better marker than P-selectin expression on platelets due to its transient expression (51, 55). In addition, thrombotic events can be initiated and propagated through interaction between activated innate immune leukocytes (neutrophil and monocyte) and endothelial cells (e.g. between CD11b/CD18 and endothelial cell Intercellular Adhesion Molecule (ICAM)-1; chemokine receptor 2 and ligand 2) (52–54).

A targeted reduction or inhibition of these surface molecules on leukocytes using antibodies may provide an additional avenue to help reduce the incidence of thrombosis (101, 102). To further address this issue, a number of new strategies are being investigated *in vitro* and clinically such as optimization of the oxygenator PMP membrane surface (82) and administration of supplemental nitric oxide (NECTAR-KIDS randomized control trial; ACTRN12619001518156) to help restore the balance of the immunologic and hemostatic systems. Neutrophil and monocyte assessment, as well as their extracellular vesicle thrombotic phenotype may also prove to be beneficial in these studies.

## Future Directions

Current evidence underscores the importance of leukocytes and their subpopulations as biomarkers of inflammation, predictors of mortality and/or poor patient outcomes across a spectrum of critical illnesses (56, 63, 76, 103, 104). There have been numerous therapeutic interventions targeting leukocytes (102, 105, 106). However, available reports on leukocytes in the context of ECMO are rudimentary. This limits the potential for further technological solutions or targeted treatments to minimize ECMO-mediated leukocyte modulation, and the adverse events that accompany it. In addition to phenotypic and functional profiling, interrogation into the impact of ECMO on leukocyte extracellular vesicle generation, and their interaction with platelets and endothelial cells will be essential for knowledge progression. Future studies should also aim to address two major challenges of current leukocyte research: ECMO technological disparity and the lack of understanding in the compound effect of ECMO with other clinical stressors and therapies.

### Addressing Technological (Circuit Designs) Disparities

The technology employed in experimental and clinical ECMO research conducted pre- and post-2007 is substantially different, necessitating a technological disclaimer when performing comparative meta-analysis. Additionally, consistent reporting of the ECMO system or circuit biomaterial used in future publications would be advantageous to assist analysis. While the biomaterial and centrifugal pump between different clinically used ECMO systems are relatively homogenous at present, the membrane configuration of the oxygenators remains quite different in some. These differences may yield diverse modulation of leukocyte profiles, and similarly for other blood components (24, 30, 31, 83), however this has yet to be thoroughly investigated. In order to address the modern era ECMO technological discrepancies, a benchmark oxygenator study would prove beneficial. This may be achieved by profiling and assessing leukocyte changes and other cellular functions when exposed to different ECMO circuit designs using PMP hollow-fiber membranes. This will allow for fair comparison between studies and will help to build a more comprehensive library of immune profiles for ECMO, and future optimization of the technology.

### Understanding Factors Contributing to ECMO-Related Leukocyte Modulation

Other than the discussed non-physiological stressors of changing flow dynamics and the associated shear stress generated within the circuit, patients are also frequently exposed to therapies such as multiple blood transfusions when treating bleeding complications (107), heparinization to reduce clot formations (11, 12), supranormal administration of supplemental oxygen concerning hypoxia (108), and renal replacement therapy for ECMO-related AKI (9). These therapies have been independently reported to modulate leukocyte phenotype and function (44–46, 100, 109–111), which can result in a worsened prognosis. The cumulative impact of these therapies and stressors is likely to be significant. This provides the impetus for the development of a comprehensive mechanistic understanding of immunobiology and pathophysiology in the context of ECMO. Together, these aspects will need to be controlled in both scientific and clinical studies to make these research outcomes meaningful.

### Potential Interventions and Targets for ECMO-Related Leukocyte Modulation

Given that one of the reasons for the modulated leukocyte profiles is likely initiated by the direct contact between patient blood and the non-endothelial surfaces of ECMO circuits, components with the capacity to reduce leukocyte activation and adhesion, or to boost pathogenic function, may be beneficial. Nitric oxide is an element capable of enhancing the anti-inflammatory capacity of the immune system as well as reducing cellular activation and adhesion (112–114). An early *ex vivo* ECMO study has also highlighted the potential importance of delivery methods when using supplemental gas to improve biological and clinical outcomes (e.g. direct infusion into circulating blood *versus* administrating into the oxygenator) (33). The benefit of nitric oxide administration into the oxygenator in patients undergoing cardiac surgery has been demonstrated with improved outcomes in a number of randomized control trials (115, 116). Further, adoption of specific leukocyte targets may also prove more effective. For example, use of inhibitors to dampen or transiently eradicate the inflammatory response mediated by leukocyte activation and adhesion. The β_2_-integrin CD11b/CD18 surface antigen is commonly used to characterize activation of neutrophils and has recently been proposed as a novel therapeutic target for controlling inflammation (101). Thus, inhibition of the surface antigen and downstream processes may alleviate patients’ resistance to sustained manifestations of neutrophil activity, interaction with endothelial cells and migration to organs resulting in MODS. Other studies have reported efficacy in blocking adhesion surface antigens of lymphocytes using antibodies and antagonists in various inflammatory diseases (102). Alternatively, the leukocyte inhibition module, a medical device that limits overshooting of neutrophils post-operative activity following CPB, could be useful for ECMO patients. The efficacy and safety of this device has been demonstrated in patients, with the capacity to limit neutrophil activity and inflammatory responses (117–119). However, until a better understanding of leukocytes in ECMO is available, attempts to create specific immune interventions to improve patient outcomes must be cautious and will remain challenging.

## Conclusions

Leukocytes are central to the pathophysiology of critical illness. An increasingly detailed understanding of phenotypic and functional alterations in leukocyte populations during critical illness has opened new areas of research. This is less well developed in the context of ECMO, compared to other extracorporeal devices, where research is lacking. In adults particularly, no mechanistic data is available, despite the widespread adoption of this modality. If advances in ECMO technology are to translate into continued improvements in outcome this deficit needs to be addressed. Specifically, we need to understand the full spectrum of ECMO-related leukocyte modulation in the heterogenous patient populations, and that associated with different ECMO circuits and therapies. This knowledge could provide data to help delineate the immunologic signatures between primary disease burden and ECMO, optimize ECMO design, and lessen its effects on leukocytes to help reduce the frequently reported complications associated with ECMO. Equally, a careful selection of primary measures and appropriately powered clinical study design are essential in determining the impact of these biological perturbations on patient outcomes. With these insights, we may then facilitate risk stratification for patients to inform clinical decision-making and to optimize survival, quality of life, and resource allocation.

## Author Contributions

KK and JS conceived the review, and KK wrote the original draft of the manuscript. JM, DL, MP, CM, KS, MS-H, HC, JS, and JF reviewed the manuscript, and rewrote individual sections. Both JS and JF have contributed equally to this manuscript as co-last authors. All authors contributed to the article and approved the submitted version.

## Funding

Publication of this review is funded by The National Health and Medical Research Council Centre for Research Excellence in Advanced Cardio-respiratory Therapies Improving OrgaN Support (APP1079421, Australia) and The Prince Charles Hospital Foundation (The Common Good). This work was also supported by the National Institute for Health Research Clinician Scientist Award (CS-2016-16-011 for MS-H), the Metro North Hospital and Health Service, University of Queensland and Queensland Health Bionics (for KK), Advance Queensland (for JS), and the Office of Health and Medical Research, Queensland Health (for JF).

## Conflict of Interest

The authors declare that the research was conducted in the absence of any commercial or financial relationships that could be construed as a potential conflict of interest.
