# Supplementary material for: Current Understanding of Leukocyte Phenotypic and Functional Modulation During Extracorporeal Membrane Oxygenation: A Narrative Review
Source: Front Immunol. 2021 Jan 8;11:600684. doi: 10.3389/fimmu.2020.600684 (PMC7821656; doi:10.3389/fimmu.2020.600684)
Supplement: Supplementary file 1 [file DataSheet_1.pdf]

Supplementary Material

Supplemental Literature Search Strategy

Data sources: Online search of EMBASE and PubMed/MEDLINE for all relevant publications was conducted. Search terms were 'extracorporeal membrane oxygenation' OR 'ECMO' OR 'extracorporeal life support' OR 'ECLS' OR 'artificial heart/lung' AND 'leukocytes' OR 'white blood cells' OR 'immune response'.

Study selection and data extraction: Retrospective, randomised trials, observational and physiologic studies specifically report on leukocyte modulation during ECMO and published by 1st May 2020 were included to inform this narrative review. Non-English articles, and published abstracts that were later published into full journal articles were excluded.

Supplemental Table 1. Brief description of leukocyte activation and adhesion surface antigens/markers discussed.

Surface markers	Assessed cell type in the reported ECMO studies	Function
**CD11b**	Neutrophils	Part of the integrin family, essential to regulating leukocyte adhesion to the endothelium and migration. This mediates the inflammatory response and cell infiltration to the site of injury or infection as part of early host immune defence. It is co-expressed with CD18 (β_2-integrin CD11b/CD18), the complex is known as MAC-1 and CR3. It also interacts with platelets glycoprotein and fibrinogen that triggers thrombogenicity (1, 2).
**CD18**	Neutrophils, monocytes, T and B lymphocytes	Part of the integrin family, essential to regulating leukocyte adhesion to the endothelium and migration. It is co-expressed with CD11b (β_2-integrin CD11b/CD18), the complex is known as MAC-1 and CR3. It also interacts with platelets glycoprotein and fibrinogen that triggers thrombogenicity (1, 2).
**CD35**	Neutrophils	Also known as CR1 that binds complement 3b and 4b, essential for clearance of foreign macromolecules. It can also differentially regulate a number of TLR responses, and regulate B lymphocyte function (3).
**CD62L**	Neutrophils, monocytes, T and B lymphocytes	Adhesion molecule part of the selectin family, essential to leukocyte trafficking from the circulation to the tissue (4).
**CD142**	Monocytes	Also known as tissue factor which ligands are Factor VIIa, and Factor Xa/TFPI. Essential for initiation of pro-thrombotic process for clotting (5).
**CD161**	T lymphocytes	Part of the C-type lectin receptor family. It is known to express on specific peripheral T lymphocyte subsets, which regulates IL-17 production (6).
**HLA-DR**	Monocytes	Also known as the MHC-II, essential for initiation of immune response. It is a peptide-loading protein for presentation to T lymphocytes acting as a bridge between innate and adaptive immune communications (7).
**TLR4**	Monocytes	Part of the pattern-recognition family, essential in the innate immunity. It is widely known for its recognition of exogenous pathogens (e.g. bacterial components - lipopolysaccharide and lipoteichoic acids) to fight infection, but it also recognises other endogenous molecules (e.g. fibronectin type III extra domain A) (8).

Abbreviations: CD, Cluster differentiation; CD62L, L-selectin; CR, Complement Receptor Type; HLA-DR, human leukocyte antigen DR; MHC-II, IL, Interleukin; MAC-1, Macrphage-1 antigen; Major histocompatibility complex class II; MMP, matrix metalloprotease; TLRs, Toll-like receptor

Supplemental References

1. Lishko, V.K., Yakubenko, V.P., Ugarova, T.P., and Podolnikova, N.P. Leukocyte integrin Mac-1 (CD11b/CD18, α(M)β(2), CR3) acts as a functional receptor for platelet factor 4. *J Biol Chem* (2018) 293:6869-82.DOI: 10.1074/jbc.RA117.000515.
2. Arnaout, M.A. Biology and structure of leukocyte β (2) integrins and their role in inflammation. *F1000Research* (2016) 5:F1000 Faculty Rev-2433.DOI: 10.12688/f1000research.9415.1.

3. Mácsik-Valent, B., Nagy, K., Fazekas, L., and Erdei, A. Complement Receptor Type 1 (CR1, CD35), the Inhibitor of BCR-Mediated Human B Cell Activation, Differentially Regulates TLR7, and TLR9 Induced Responses. *Front Immunol* (2019) 10.DOI: 10.3389/fimmu.2019.01493.
4. Ivetic, A., Hoskins Green, H.L., and Hart, S.J. L-selectin: A Major Regulator of Leukocyte Adhesion, Migration and Signaling. *Front Immunol* (2019) 10.DOI: DOI: 10.3389/fimmu.2019.01068.
5. Grover, S.P., and Mackman, N. Tissue Factor: An Essential Mediator of Hemostasis and Trigger of Thrombosis. *Arterioscler Thromb Vasc Biol* (2018) 38:709-25.DOI: 10.1161/ATVBAHA.117.309846.
6. Fergusson, J.R., Fleming, V.M., and Klenerman, P. CD161-expressing human T cells. *Front Immunol* (2011) 2:36-36.DOI: 10.3389/fimmu.2011.00036.
7. Cruz-Tapias, P., Castiblanco, J., and Anaya, J. (2018). "Major histocompatibility complex: Antigen processing and presentation", in: *Autoimmunity: From Bench to Bedside [Internet]*. (ed.) S.Y. Anaya Jm, Rojas-Villarraga a, Et Al. (Bogota (Colombia): El Rosario University Press).
8. Vaure, C., and Liu, Y. A comparative review of toll-like receptor 4 expression and functionality in different animal species. *Front Immunol* (2014) 5:316-16.DOI: 10.3389/fimmu.2014.00316.
